# Factor D Inhibition Blocks Complement Activation Induced by Mutant Factor B Associated With Atypical Hemolytic Uremic Syndrome and Membranoproliferative Glomerulonephritis

**DOI:** 10.3389/fimmu.2021.690821

**Published:** 2021-06-10

**Authors:** Sigridur Sunna Aradottir, Ann-Charlotte Kristoffersson, Lubka T. Roumenina, Anna Bjerre, Pavlos Kashioulis, Runolfur Palsson, Diana Karpman

**Affiliations:** ^1^ Department of Pediatrics, Clinical Sciences Lund, Lund University, Lund, Sweden; ^2^ Centre de Recherche des Cordeliers, INSERM, Sorbonne Université, Université de Paris, Paris, France; ^3^ Division of Pediatric and Adolescent Medicine, Oslo University Hospital, Oslo, Norway; ^4^ Institute of Clinical Medicine, University of Oslo, Oslo, Norway; ^5^ Department of Molecular and Clinical Medicine/Nephrology, Institute of Medicine, Sahlgrenska Academy, University of Gothenburg, Gothenburg, Sweden; ^6^ Landspitali - The National University Hospital of Iceland, Reykjavik, Iceland; ^7^ Faculty of Medicine, School of Health Sciences, University of Iceland, Reykjavík, Iceland

**Keywords:** complement, factor B, factor D, danicopan, atypical hemolytic uremic syndrome, C3 glomerulopathy

## Abstract

Complement factor B (FB) mutant variants are associated with excessive complement activation in kidney diseases such as atypical hemolytic uremic syndrome (aHUS), C3 glomerulopathy and membranoproliferative glomerulonephritis (MPGN). Patients with aHUS are currently treated with eculizumab while there is no specific treatment for other complement-mediated renal diseases. In this study the phenotype of three FB missense variants, detected in patients with aHUS (D371G and E601K) and MPGN (I242L), was investigated. Patient sera with the D371G and I242L mutations induced hemolysis of sheep erythrocytes. Mutagenesis was performed to study the effect of factor D (FD) inhibition on C3 convertase-induced FB cleavage, complement-mediated hemolysis, and the release of soluble C5b-9 from glomerular endothelial cells. The FD inhibitor danicopan abrogated C3 convertase-associated FB cleavage to the Bb fragment in patient serum, and of the FB constructs, D371G, E601K, I242L, the gain-of-function mutation D279G, and the wild-type construct, in FB-depleted serum. Furthermore, the FD-inhibitor blocked hemolysis induced by the D371G and D279G gain-of-function mutants. In FB-depleted serum the D371G and D279G mutants induced release of C5b-9 from glomerular endothelial cells that was reduced by the FD-inhibitor. These results suggest that FD inhibition can effectively block complement overactivation induced by FB gain-of-function mutations.

## Introduction

The innate immune system is a first line of defense against pathogens and contributes to removal of apoptotic host cells. One of the mainstays of protection is the complement system responding to non-self molecules and eliminating them or neutralizing their effects by opsonization or lysis as well as induction of leukocyte chemoattraction, inflammation and phagocytosis (1). The main enzymatic activity of the alternative complement pathway is mediated by the C3 convertase. For C3 convertase formation to occur C3b binds to factor B (FB) which is cleaved by factor D (FD) to the Ba and Bb fragments, the latter possessing catalytic activity. Bb remains bound to C3b and forms the C3bBb convertase that exponentially cleaves more C3 into C3a and C3b. Binding of additional C3b molecules generates the C5 convertase. The complement system is kept in balance by multiple cellular and soluble regulators (1).

FB is essential for defense against encapsulated bacteria, and thus individuals with FB deficiency are prone to infection with *Neisseria meningitidis* and *Streptococcus pneumoniae* (2). Conversely, an overactive FB can lead to excessive complement activation *via* the alternative pathway resulting in kidney diseases such as atypical hemolytic uremic syndrome (aHUS) or C3 glomerulopathy. In both of these rare conditions, patients may exhibit complement activation but there are distinct differences in clinical presentation and renal pathology. While aHUS is characterized by hemolytic anemia, thrombocytopenia and renal failure with lesions indicative of thrombotic microangiopathy (3), C3 glomerulopathy is a form of chronic glomerulonephritis presenting with hematuria and proteinuria leading to renal failure (4). These conditions can arise due to mutant variants in complement factors, including C*FB* mutations, or auto-antibodies against factor H (5). Autoantibodies against FB have been described in C3 glomerulopathy (6).

In aHUS the complement system is overactive due to loss-of-function mutations in complement regulators or gain-of-function mutations in *C3* or *CFB* (7). Gain-of-function variants in *CFB* are rare and have in certain cases been associated with low C3 levels in patient sera (8–11) indicating complement activation *in vivo*. Mutations have been shown to increase FB binding affinity to C3b thereby stabilizing the C3bBb convertase (12) and enhancing resistance to factor H mediated decay acceleration (9, 13). This was particularly demonstrated for mutations located in close proximity to the C3b binding region, i.e. the Mg^2+^-binding site in the von Willebrand factor type A domain of FB (14). Of note, not all *CFB* mutations have been shown to induce complement activation and not all individuals carrying *CFB* mutations associated with aHUS develop disease (11, 12, 14), even if circulating C3 levels are low. In addition to aHUS, *CFB* mutations and rare variants have also been demonstrated in C3 glomerulopathy and immune complex-associated membranoproliferative glomerulonephritis (MPGN) (15–17).

Binding of C3b to FB elicits a conformational change exposing the scissile bond at position Arg^234^-Lys^235^ enabling cleavage by FD (18). Small molecule FD inhibitors have been developed as potential treatments for complement-mediated diseases (19) and efficiently inhibited activation of the alternative pathway *in vitro* as well as in animal models (19, 20). FD inhibitors present the advantage of blocking complement activation at the level of the C3 convertase, while leaving the classical and lectin pathways intact. A phase 2 trial has been completed and a phase 3 trial with an oral FD inhibitor as an add-on therapy to C5 inhibition is ongoing in patients with paroxysmal nocturnal hemoglobinuria (PNH) (21, 22).

The aim of this study was to investigate if FD inhibition impacted complement overactivation induced by *CFB* mutations. To this end we investigated four *CFB* mutations associated with aHUS or MPGN, two of which mediate a gain-of-function phenotype. We studied the effect of FD inhibition in the presence of the FB mutations on C3 convertase-induced FB cleavage, complement-mediated hemolysis, and release of soluble C5b-9 from glomerular endothelial cells.

## Materials and Methods

### Subjects

Patients from Iceland, Sweden and Norway with complement-mediated renal diseases are referred to the laboratory at the Dept of Pediatrics in Lund for genetic diagnostics. Three patients were found to have *CFB* mutations. The patients and their laboratory data are presented in **Table 1**. Samples were obtained from apparently healthy adult controls (n=12, 6 female) who were not using any medications. The study of patients and healthy controls was performed with the approval of the Ethics Review Board at Lund University. Approval included genetic analysis of Nordic patients and phenotypic studies of complement mutations. The study was also approved by the National Bioethics Committee of Iceland and the Data Protection Officer at Oslo University Hospital, Oslo Norway. Informed written consent was obtained from the patients or the parents of Patient 3 and the healthy controls.

**Table 1:** Clinical characteristics of patients included in this study.

Pat	Sex	Age at presentation (yrs)	Diagnosis	Clinical presentation	Biopsy findings	Disease course	Complement levels		Genetic assay^a^			
							C3 g/L	Factor B %	Factor B	Factor H	DGKE	ADAMTS13
1	M	1	aHUS	Uremia	TMA	CKD stage 5	0.4	100	D371G	–	–	–
				Hypertension	C3 deposition	Eculizumab						
						Recurrences: 4						
						Kidney transplant x3^b^						
2	F	54	aHUS	Uremia	NA	CKD stage 3	0.9	66	E601K	–	Q560R	–
						Eculizumab						
						Recurrence 0						
3^c^	F	6	MPGN	Nephrotic syndrome	MPGN	CKD stage 5	0.75	79	I242L	V62I N1050Y	–	P457L
					C3, C1q, C5b-9 and IgM deposition	Eculizumab						

^a^All genetic variants shown are heterozygous. ^b^Two kidney transplants were performed before the eculizumab era, the first functioned for 15 years and the second for 7 years. Three years after the second transplant, treatment with eculizumab was initiated due to HUS recurrence and was continued until the patient returned to dialysis. Eculizumab therapy was restarted at the time of the third kidney transplant without evidence of recurrence. ^c^This patient underwent two biopsies within 2 months, C3 deposits in capillary walls and mesangium increased in the second biopsy. The patient did not have circulating C3 nephritic factor. DGKE, diacylglycerol kinase epsilon; aHUS, atypical hemolytic uremic syndrome; TMA, thrombotic microangiopathy; CKD, chronic kidney disease; MPGN, membranoproliferative glomerulonephritis; NA, not available/not performed. Normal reference values for C3: 0.5-0.95 g/L (Patient 1) 0.77-1.38 g/L (Patients 2 and 3), reference values vary between different clinical laboratories; Factor B: 75-125% (Patient 1), 59-154% (Patients 2 and 3). Patients 1 and 2 did not have antibodies against factor H.

### Blood Samples

Whole blood in EDTA tubes was used for DNA purification. Serum samples were taken during chronic disease in Patients 1-3 and from healthy controls, centrifuged after one hour at room temperature and stored at -80°C until assayed.

### Genetic Analysis and Mutation Screening

Next generation sequencing was performed focusing on a panel of genes encoding the following 17 proteins: complement C3, CFB, factor H (CFH), factor H-related proteins-1, -2, -3, -4, -5, C5, factor I, properdin, CD46 (membrane co-factor protein), a disintegrin and metalloproteinase with a thrombospondin type 1 motif 13 (ADAMTS13), diacylglycerol kinase epsilon (DGKE), plasminogen, thrombomodulin and clusterin.

Whole-exome sequencing was performed at the Center for Molecular Diagnostics, Skåne University Hospital and Clinical Genomics Lund, SciLifeLab. In brief, genomic DNA was subject to tagmentation-based library preparation and hybrid capture using the Illumina TruSeq Rapid Exome library kit according to the manufacturer’s instructions. Captured exome libraries were sequenced 2 x 150 bp on a Next Seq 500 (Illumina, San Diego, CA). Alignment and variant calling were performed according to GATK “Best Practices for Germline SNP & Indel Discovery in Whole Genome and Exome Sequence”. Sequence data was mapped to the hg19 genome build using BWA 0.7.15 (23) and variants were called using HaplotypeCaller from GATK 3.7 and processed according to the best practice recommendations (24, 25). Variants were annotated using Ensembl Variant Effect Predictor release 87. Reported variants with frequencies <2% according to Genome Aggregation Database (gnomAD) were included in the genetic evaluation.

### Measurement of Anti-Factor B Antibodies

Factor B antibodies were measured in serum as previously described (6). A Nunc Maxisorp 96 well plate (Thermo Scientific, Roskilde, Denmark) was coated with FB (Complement Technologies, Tylor, Texas). Bound IgG was detected with anti-human IgG:horse radish peroxidase (DAKO, Glostrup, Denmark).

### Mutagenesis

A plasmid containing wild-type *CFB*, or variants D279G, or I242L cDNA in the expression vector, pcDNA 3.1/V5-His TOPO (Invitrogen, Thermo Fisher, Carlsbad, CA) was previously described (12, 26). For the FB variants D371G and E601K mutagenesis was performed using the QuikChange II XL Site-Directed Mutagenesis Kit (Agilent Technologies, Santa Clara, CA). Primers are available upon request. Mutant sequences were verified by enzymatic digestion with restriction enzymes. XL-10 Gold ultracompetent cells (Agilent) were used for transformation. Constructs were Sanger sequenced to confirm that no additional mutations had been introduced.

### Transient Transfection

Transfection was performed as previously described (27). Briefly, human embryonic kidney 293 cells (HEK) cells (ATCC, Teddington, Middlesex, UK) were seeded and grown in DMEM/high glucose Hyclone medium (GE Healthcare Life Sciences, South Logan, UT), supplemented with 100 U/mL penicillin, 100 μg/mL streptomycin and 10% fetal bovine serum to approximately 95% confluence before transfection. Plasmid DNA (2 μg) was added to each well and transfection performed with Lipofectamine (Invitrogen, Life Technologies, Waltham, MA) according to the manufacturer’s instructions. Twenty-four h after transfection, the medium was changed to Optimem (Thermo Fisher Scientific) and cells were cultured for an additional 72 h. The media were collected and supplemented with protease inhibitors cOmplete Mini without EDTA (Roche Diagnostic, Mannheim, Germany) and centrifuged to remove cell debris.

### Determination of Factor B Size by Immunoblotting

FB size in sera and cell media was determined by immunoblotting. Sera was diluted 1:2000, samples were reduced with mercapto-ethanol and incubated at 100°C for 5 minutes. Proteins were separated by SDS electrophoresis and transferred to a PVDF membrane. Plasma purified FB (1 mg/mL, Complement Technology, Tyler, Texas) was used as the control. Membranes were blocked overnight. Polyclonal goat anti-human FB antibody (1:1000, Complement Technology) was used as the primary antibody followed by rabbit anti-goat horse-radish-peroxidase (1:1000, DAKO, Glostrup, Denmark). Detection was performed by chemiluminescence (Pierce lECL2, Western Blotting Substrate, Rockford, IL) and detected using ChemiDoc™ Touch, Bio-Rad (Hercules, CA).

### Measurement of Factor B Protein Levels

FB concentration of constructs was quantified by ELISA using mouse anti-human factor Ba (Quidel, San Diego, CA) for capture and goat anti-human FB polyclonal antibody (Complement Technology) for detection, followed by rabbit anti-goat horse-radish-peroxidase (HRP, 1:1000, DAKO, Glostrup, Denmark), alternatively an ELISA kit for detection of human FB (Abcam, Cambridge UK) showing comparable FB levels. Plasma purified FB was used as the standard. Absorbance was measured at 450 nm using Glomax Discover (Promega, Madison, WI).

### Complement Activation on Primary Glomerular Endothelial Cells

Primary glomerular endothelial cells (Cell Systems, Kirkland, WA) were plated on cell culture slides (Thermo Fisher Scientific) in endothelial growth medium (EGM-2, Lonza, Walkersville, MD), approximately 75000 cells per well and cultured to monolayer confluence. Cells were activated with adenosine diphosphate (ADP, 1 mM, Sigma-Aldrich) in serum-free EGM-2 for 10 min and washed with PBS with Mg/Ca (GE Life Sciences). Serum samples were diluted 1:4 in serum-free EGM-2 and magnesium-ethylene glycol-bis(2-aminoethylether)-N N N´N`-tetraacetic acid (Mg-EGTA, Complement Technology) 0.1M 1:10, incubated with the cells for 2 h at 37°C. Cells were washed, fixed in paraformaldehyde 4% for 30 min, washed thrice and blocked in 1% BSA for 30 min. C3c deposition on the cells was detected using rabbit anti-human C3c antibody:FITC (DAKO, Glostrup, Denmark) diluted 1:50 in 1% BSA for 1 h. Cells were stained with 4´,6-diamidino-2-phenylindole (DAPI, Thermo Fisher, Eugene, OR). Fluorescence was detected using a Ti-E inverted fluorescence microscope equipped with a Nikon structured illumination microscopy module (Nikon Instruments, Tokyo, Japan). Image stacks at 10 x magnification were converted to maximal intensity images. Stained cells were outlined with a threshold above the background to select the area occupied by cells (DAPI-positive). Quantification was performed using ImageJ Fiji Software (Version 1.53h, NIH, Bethesda, MD).

In certain experiments the cells were incubated with FB constructs (50 μg/mL) in FB-depleted serum diluted 1:4 in serum-free EGM-2. The FB constructs were preincubated with and without the FD inhibitor danicopan ACH- 4471 (MedChemtronica AB, Monmouth Junction, NJ) 10 μM for 15 min before a 2-hour incubation with the cells. Cell supernatants were collected and kept at -20°C until assayed using the sC5b-9 ELISA described below.

### Hemolytic Assays Using Human Sera and Factor B Constructs

Complement activation in serum was assayed by incubation of the serum with sheep erythrocytes (5x10^8^/mL, Håtunalab, Bro, Sweden). Serum (20%) was added to gelatin veronal buffer (GVB) with Mg-EGTA 0.1M 1:10 to which normal human serum (20%) was added, as a source of normal C3, for 10 min at 30°C (28). Ethylenediaminetetraacetic acid (EDTA) 10 mM was added, and samples were centrifuged. Rat serum in EDTA (1:5, Complement Technology) was added, as a source of the terminal complement pathway, for 1 h at 37°C and samples were centrifuged. Absorbance was measured at 405 nm using Glomax Discover (Promega, Madison, WI).

Rabbit erythrocytes (5x10^8^/mL, Håtunalab) were used in GVB- Mg-EGTA buffer, as above, and incubated with FB constructs 5 μg/mL in FB-depleted serum (Complement Technology). Samples were incubated for 1 h on a shaker at 37°C, after which complement activation was terminated by addition of EDTA (Complement Technology). In certain experiments the FD inhibitor was incubated with erythrocytes in buffer to which FB-depleted serum was added before addition of the constructs. Absorbance was measured at 405 nm using Glomax Discover.

### Binding of Factor B Constructs to C3 Measured by Surface Plasmon Resonance

Purified C3b (33 μg/mL in 10 mM sodium acetate (GE Healthcare Bio-Sciences), pH 5.0, Complement Technology) was amine-coupled to a CM5 sensor chip (GE Healthcare) corresponding to 5517.3 response units. The surface of the sensor chip was activated with a mixture of N-hydroxysuccinimide and 1-ethyl-3-(3-dimethylaminoipropyl) -carbomide. After covalent binding of C3b to the dextran matrix the surface was blocked with ethanolamine. Running buffer (10 mM Hepes (pH 7.4), 50 mM NaCl, 10 mM MgCl_2_) was injected over the flow cells at a flow rate of 10 μL/min and 25°C. The C3 convertase was generated as previously described (29) by serial injections of FB 0.28 µg (60 nM) together with FD 0.02 µg in 50 µl running buffer followed by C3(H2O) 2 µg. After the last step (FB+FD) the surface was extensively washed with 3 M NaCl in acetate buffer (pH 5.2) and 50 mM NaOH to rinse away residual noncovalently bound proteins.

Additional experiments were performed to study the interaction between C3b and FB. C3b was diluted in 10 mM sodium acetate (pH 5.5) at a concentration of 20 μg/mL, and then immobilized as above. The D371G, D279G and wild-type constructs were injected at a flow rate of 30 μL/min and at 25°C over the flow cells using a running buffer containing 10 mM Hepes, 150 mm NaCl, 0.005% surfactant P20, 3.4 mM EDTA (pH 7.5) and 10 mM MgCl_2_. The equilibrium constant (KD) was calculated. The regeneration buffers used were 10 mM glycine-HCl pH 1.5 and 0.5 M sodium chloride.

Assays were performed using a Biacore X100 instrument (GE Healthcare). Biacore X100 Evaluation Software version 2.0.1 and 1.0.1 was used for sensogram generation. Baseline values were adjusted to pre-sample injection levels at *t*=0 in each cycle in order to compare binding.

### Factor B Cleavage by Factor D in Solid Phase

Microtiter wells were coated with C3b at 3 μg/mL diluted in phosphate-buffered-saline (PBS) overnight and blocked with bovine serum albumin (BSA, Sigma) for 1 h. Serum, 20% in Mg- PBS, or FB constructs 5 μg/mL in assembly buffer (Mg, FD 100 ng/mL in PBS with BSA 1%), were incubated for 30 min at 37°C and an additional 30 min with slow shaking. Samples were washed four times with PBS-Tween 20 0.1%. Twenty μl 10 mM EDTA with sodium dodecyl sulfate (SDS) 1% were added to the empty wells for 1 h on a microplate shaker 1000 rpm at rt. Protein complexes were detached by scraping as described (30) and samples were stored at -20°C. In certain samples the FD inhibitor at 10 μM (final concentration) was preincubated with the samples for 15 min before addition to the plate.

Samples were reduced and loaded onto a Tris-TGX gel 10% (Bio Rad) and after protein separation transferred to PVDF membranes (Bio Rad) and electroblotted (Transblot Turbo, Bio Rad). Immunoblot was carried out with goat anti-FB and rabbit anti-goat:HRP and detected as described above.

### Soluble C5b-9 Measurement

Soluble C5b-9 in the supernatant from activated glomerular endothelial cells was quantified using the MicroVue SC5-b9 Plus kit (Quidel, San Diego, CA) according to the manufacturer’s protocol. Absorbance was measured at 450 nm using Glomax Discover.

### Statistics

Kruskal-Wallis multiple-comparison test followed by Dunn’s procedure was used for evaluating differences between more than two groups. A P value ≤ 0.05 was considered significant. Statistical analysis was performed using GraphPad prism 8 software (version 8.4.3, GraphPad Software, La Jolla, CA).

## Results

### Factor B Variants

**Figure 1:**
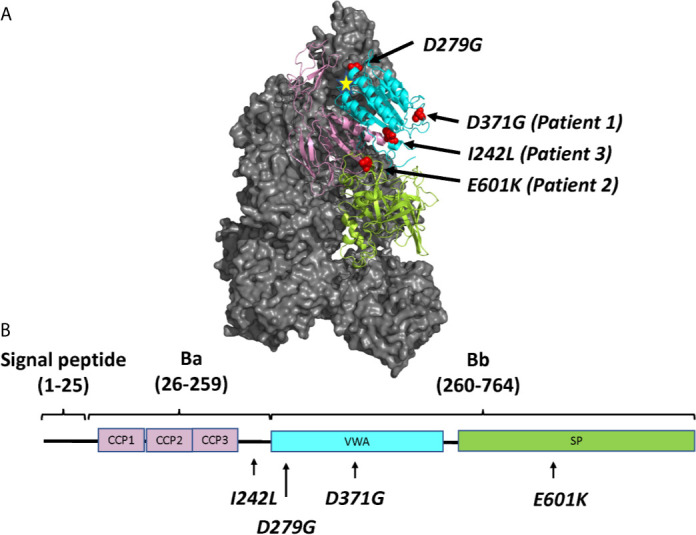
The molecular structure of factor B and the location of mutations described in this study. **(A)** Location of the four mutated residues (D371G, E601K, I242L and D279G), visualized on the structure of the C3 pro-convertase C3bB depicted in grey (PDB ID 2XWJ) (18) using PyMol. The Metal Ion Dependent Adhesion Site (MIDAS) on C3b is depicted by a star. The colors of the domains correspond to the domains depicted in **(B)**. **(B)** The linear structure of factor B divided into the signal peptide/leader, Ba and Bb fragments with a linker sequence in between. The amino acid numbers are given in parentheses. The protein is composed of a complement control protein (CCP) domain with three CCPs, followed by the linker, the von Willebrand factor type A domain (VWA) and the serine protease (SP) domain. The location of mutations studied herein is depicted.

Three FB mutations were identified in Patients 1-3 (**Table 1**). The location of the gene products within FB domains is depicted in **Figure 1**. FB variants D371G (rs1258425617) (31) and I242L (rs1299040443) (7, 12, 32) have been reported before in patients with aHUS. Variant D371G (rs756325732) is located in the von Willebrand factor (VWF) type A domain of the Bb fragment, but far from the C3b binding site (**Figure 1**). The E601K variant has not been previously reported and is located in the serine protease domain, not near the catalytic site, at the VWF type A domain binding interface in the context of the pro-convertase C3bB (but not in the convertase C3bBb). The Bb fragment undergoes a conformational change upon release of Ba, leading to assembly of the metal ion dependent adhesion site (MIDAS). The mutated residue is far from the MIDAS, which participates in C3b binding, but may affect its assembly by allosteric effects (**Figure 1**). I242L is located in the linker between Ba and Bb fragments near the R234-K235 scissile bond (33). The D279G variant was used as a positive control as it was previously shown to induce a gain-of-function phenotype (34) and is located in proximity to the MIDAS in the von Willebrand factor A domain. The patients had normal serum FB levels (**Table 1**) and normal FB size (**Supplementary Figure 1**) and did not have autoantibodies to FB (data not shown).

### Phenotypic Assays of the FB Mutations

Assays were performed to investigate the factor B phenotype using patient sera and mutant constructs, as outlined in **Table 2**.

**Table 2:** Complement functional assays performed in this study using patient sera and mutant constructs.

Complement assays	Patient 1		Patient 2		Patient 3		Positive control D279G	Normal controls	
	D371G		E601K		I242L				
	Serum^a^	Mutant construct	Serum^a^	Mutant construct	Serum^a^	Mutant construct	Mutant construct	Serum	Wild-type construct
C3 deposition on glomerular endothelial cells	+	ND	+	ND	+	ND	ND	3/10^b^	ND
Hemolysis sheep RBCs	+	ND	–	ND	+	ND	ND	0/2	ND
Factor B binding to C3b (surface plasmon resonance)	ND	+	ND	–	ND	–	+	ND	–
Factor B degradation by factor D	Degr	Degr	Degr	Degr	Degr	Degr	Degr	Degr	Degr
Hemolysis of rabbit RBCs	ND	+	ND	–	ND	–	+	ND	–
Soluble C5b-9 release from glomerular endothelial cells	ND	+	ND	–	ND	–	+	ND	–

^a^Serum from Patients 1 and 2 was taken during eculizumab treatment. Serum from Patient 3 was taken before the start of eculizumab treatment. ^b^3/10 indicates positive result in 3 of 10 controls. +, complement activation detected; -, complement activation was not detected; ND, Not done; RBCs, red blood cells; Degr, degradation detected.

#### C3 Deposition on Glomerular Endothelial Cells

**Figure 2:**
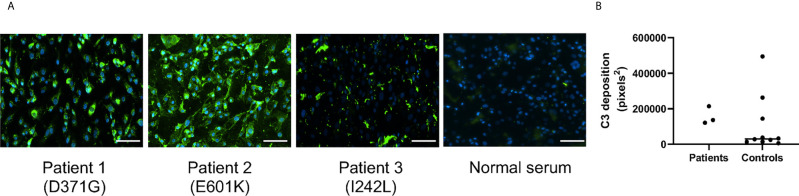
C3 deposition on glomerular endothelial cells in the presence of serum from patients and controls. Sera from Patients 1-3 and controls (n = 10) were incubated with glomerular endothelial cells for 2 h and C3 detected by immunofluorescence. **(A)** Serum from Patients 1-3 induced excessive C3 deposition on the cells. The scale bar represents 100 µm. **(B)** Quantification of C3 deposition fluorescence showing that even 3/10 sera from apparently healthy adult controls exhibited C3 deposition. The bar represents the median.

Serum from Patients 1-3 and normal sera (n=10) were incubated with primary glomerular endothelial cells. Patient sera induced C3 deposition on the cells which was also detected for 3 normal sera incubated with the cells (**Figure 2**). C5b-9 deposition on the cells could not be assayed because the patients were treated with the anti-C5 antibody eculizumab (**Table 1**).

#### Patient Sera Induced Hemolysis of Sheep Red Blood Cells

**Figure 3:**
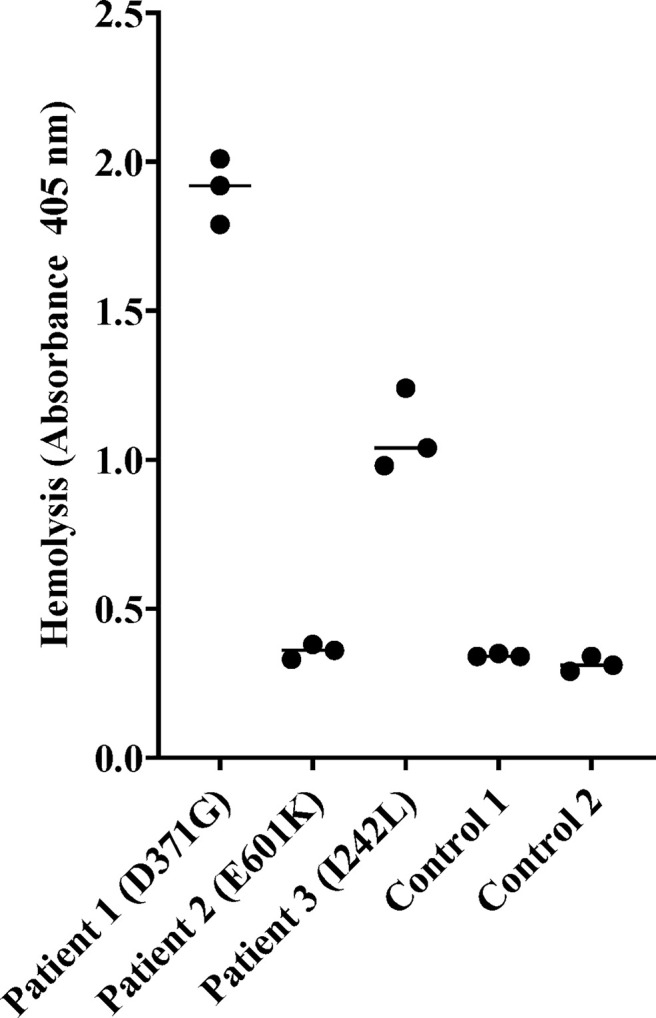
Hemolysis of sheep erythrocytes in the presence of patient and normal serum. Serum from Patients 1 (factor B mutation D371G) and 3 (I242L) induced hemolysis of sheep erythrocytes whereas serum from Patient 2 and from the two normal controls did not. The results of three separate experiments are shown. The bar depicts the median.

Sera from Patients 1-3 and normal serum from two healthy controls were incubated with sheep erythrocytes. Sera from Patients 1 (FB: D371G) and 3 (FB: I242L, CFH: V62I, N1050Y and ADAMTS13: P457L) induced hemolysis whereas samples from Patient 2 (FB: E601K, DGKE: Q560R) and the normal controls (n=2) did not (**Figure 3**).

#### Binding of Factor B Constructs to C3b and Formation of the C3 Convertase Determined by Surface Plasmon Resonance

**Figure 4:**
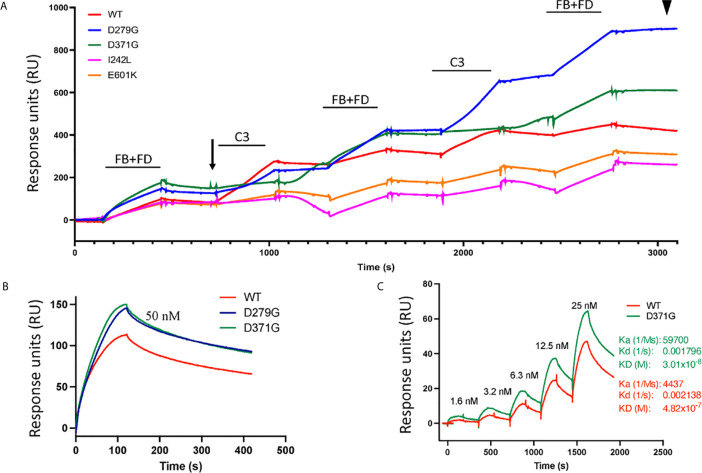
Binding of factor B variants to C3b and formation of the C3 convertase. **(A)** Purified C3b was coupled to a CM5 sensor chip. Factor B (FB) variants and factor D (FD) were injected over the surface and binding curves visualized. The FB D371G mutant exhibited the strongest binding to the C3b-coated surface (see arrow) followed by D279G, I242L, the wild-type (WT) and E601K. This was followed by serial injections of C3 alternating with FB+FD to form the C3 convertase on the chip. The strongest C3 convertase generation was demonstrated for the D279G mutant (see arrowhead), followed by D371G, the wild-type, I242L and E601K. Baseline values were adjusted at *t* = 0 in each cycle for comparison. **(B)** Binding between C3b and FB alone was assessed using the wild-type construct, D371G and D279G showing that both mutant constructs, at 50 nM, exhibited stronger binding than the wild-type construct. **(C)** The coefficient of dissociation was evaluated using a range of FB concentrations comparing construct D371G to the wild-type.

In binding assays, we first examined FB binding to C3b. C3b was immobilized on a Biacore sensor surface. The purified FB constructs, D279G (positive control, gain-of-function mutation in aHUS) (13), D371G, I242L or E601K were injected together with FD. Sensograms were aligned at *t* = 0 for comparison and showed that the FB construct D371G bound most, followed by D279G. The I242L, E601K and wild-type constructs demonstrated similar binding capacity (see arrow in **Figure 4**).

The C3 convertase was assembled on the sensor chip by serial injections of purified FB and FD followed by C3 (**Figure 4**) and showed that the factor B variant D279G yielded the highest binding, indicating C3 convertase formation, followed by D371G, the wild-type, E601K and I242L (see arrowhead in **Figure 4**).

Binding experiments showed that the FB mutant constructs D279G and D371G exhibited stronger binding to C3 than the wild-type construct (**Figure 4B**). Using a concentration range of the D371G and wild-type constructs (**Figure 4C**) the Ka, Kd and KD constant were calculated showing that the D371G construct had a higher affinity for C3 than the wild-type construct.

### Effects of Factor D Inhibition

#### Factor B Cleavage by the C3 Convertase

**Figure 5:**
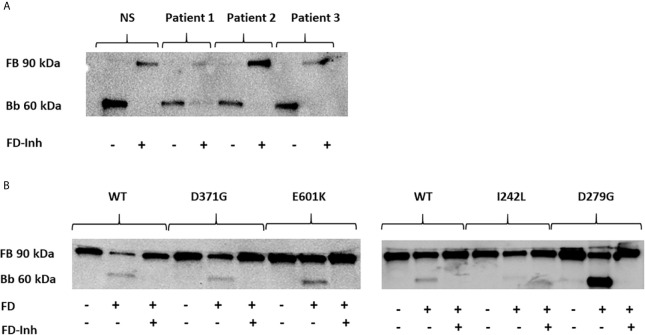
The effect of factor D inhibition on C3 convertase formation using human serum or factor B mutants. An immunoblot assay was used to detect the Bb fragment of the alternative pathway C3bBb. **(A)** C3bBb(Mg^2+^) complexes were formed by incubating C3b-coated wells with normal serum (NS) or patient serum (Patients 1-3). The C3 convertase formed in the presence of all sera effectively cleaved factor B to the Bb component and this reaction was inhibited by the factor D inhibitor. The factor D inhibitor only partially blocked the C3 convertase in the presence of serum from Patient 1(D371G mutation) as a weak Bb band was still visible. **(B)** The same assay was performed with the wild-type (WT) and mutant factor B constructs (D371G, E601K, I242L and D279G) showing cleavage to the Bb fragments and effective inhibition by the factor D inhibitor. FB, factor B; Bb, the Bb fragment of factor B; FD, factor D; FD-inh, factor D inhibitor.

A functional C3bBb(Mg^2+^) complex was formed in a microtiter plate by incubating C3b-coated wells with serum. In the presence of normal serum, as well as serum from Patients 1-3, the C3 convertase was formed and cleavage of FB to the Bb fragment was detected (**Figure 5A**). In the presence of the FD inhibitor, danicopan ACH- 4471, FB cleavage was inhibited in normal serum, as well as in the serum of Patients 2 and 3, and partially inhibited in the serum of Patient 1 in which a weak Bb band was still visible.

Similarly, the C3b-coated plates were incubated with purified FB constructs together with FD showing that all constructs, wild-type, D279G, D371G, E601K and I242L, exhibited FB cleavage to the Bb fragment, albeit weaker for the I242L variant, and that cleavage was entirely inhibited in the presence of the FD inhibitor (**Figure 5B**).

#### Complement-Mediated Hemolysis of Rabbit Red Blood Cells

**Figure 6:**
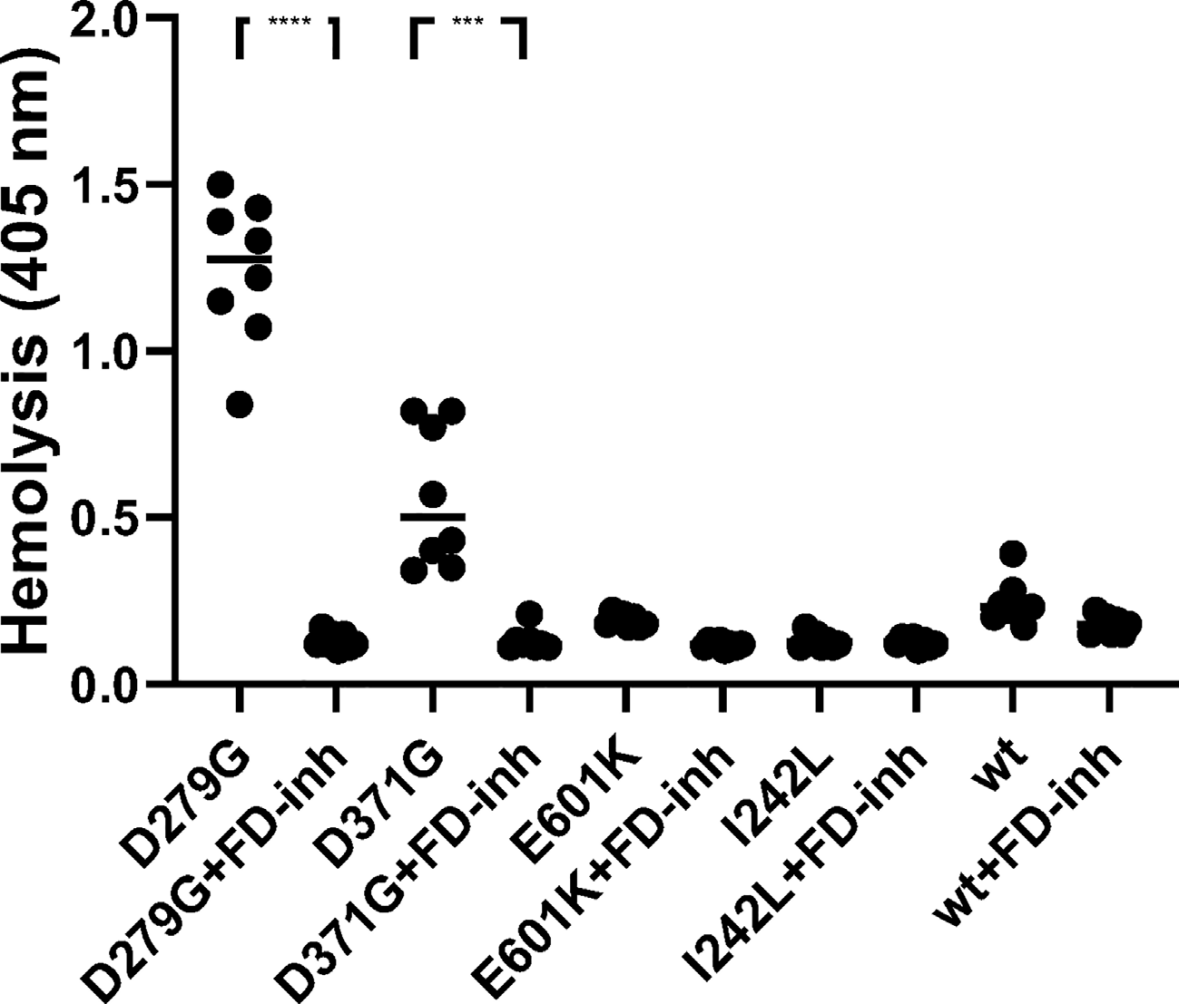
The effect of factor D inhibition on hemolysis of rabbit erythrocytes. Factor B constructs were incubated in factor B-depleted serum with rabbit erythrocytes. The mutant variants D279G (positive control) and D371G (corresponding to Patient 1) induced hemolysis. The other mutant constructs (E601K, I242L) and wild-type (wt) did not induce hemolysis. The factor D inhibitor (FD-inh) inhibited hemolysis induced by factor B mutants D279G and D371G. Eight separate experiments are shown. ***P < 0.001, ****P < 0.0001.

Rabbit red blood cells were incubated with FB constructs in FB-depleted serum and underwent hemolysis in the presence of FB variants D279G and D371G whereas the wild-type, as well as E601K and I242L constructs did not induce hemolysis. The FD inhibitor inhibited D279G- and D371G-induced hemolysis (**Figure 6**).

#### Release of Soluble C5b-9 From Primary Glomerular Endothelial Cells

**Figure 7:**
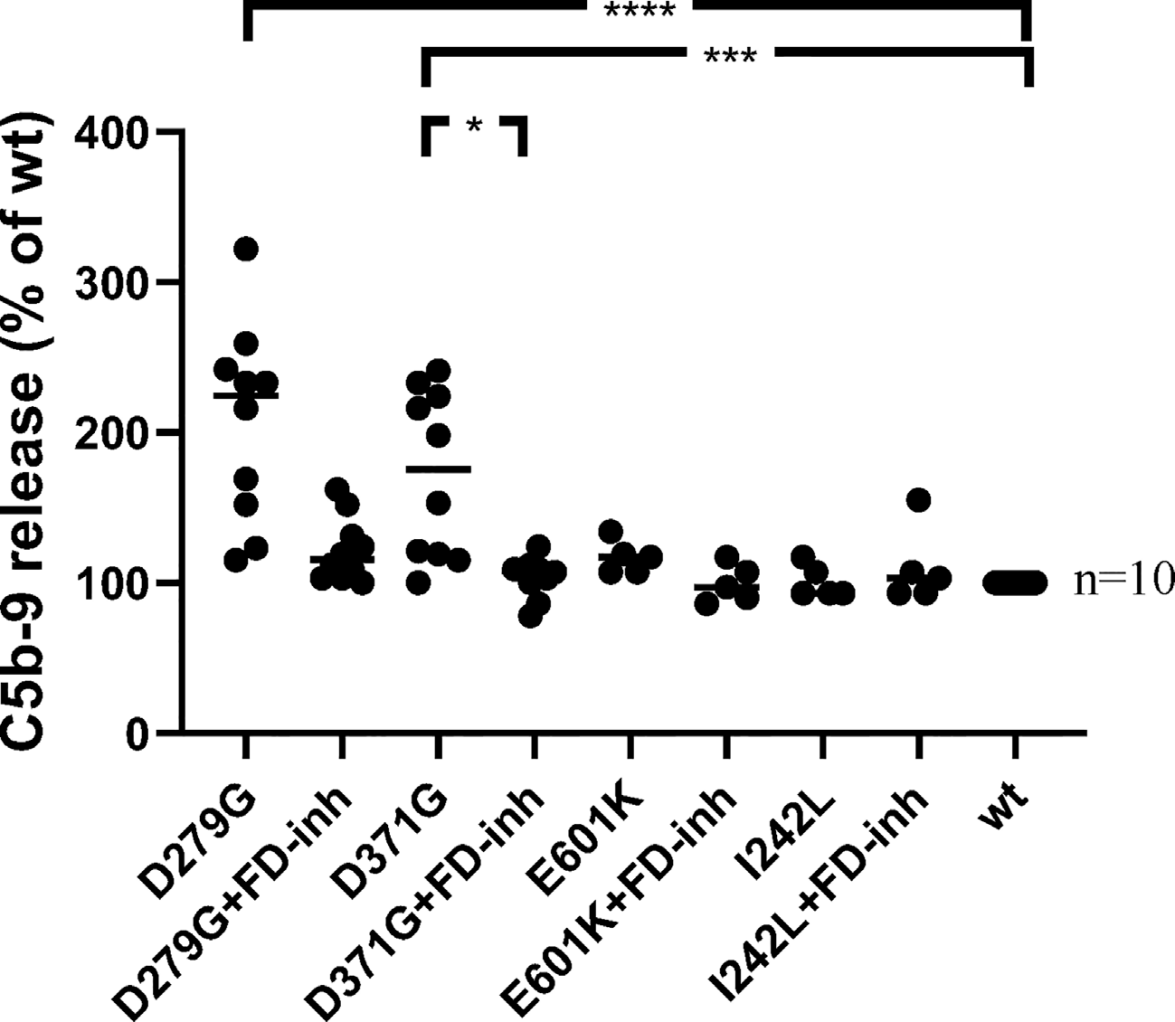
The effect of factor D inhibition on C5b-9 release from glomerular endothelial cells. Factor B mutants D279G (positive control), D371G, E601K, I242L and the wild-type (wt) construct were combined with factor B-depleted serum and incubated with glomerular endothelial cells. The release of C5b-9 was detected in cell supernatants in the presence or absence of the factor D inhibitor. The mutant construct D371G induced the release of C5b-9 which was decreased by the factor D inhibitor. A tendency to decrease was noted when the mutant construct D279G was incubated with the factor D inhibitor but this did not achieve statistical significance (multivariate analysis). FD-inh, factor D inhibitor; *P < 0.05, ***P < 0.001, ****P < 0.0001.

Soluble C5b-9 was detected in supernatants from primary glomerular endothelial cells that were incubated with FB constructs D279G and D371G in FB-depleted serum. The FB construct D371G induced increased C5b-9 release compared to the wild-type construct (**Figure 7**). In the presence of the FD inhibitor the soluble C5b-9 levels were comparable with those released in the presence of the wild-type construct (**Figure 7**). Factor B mutant constructs E601K and I242L did not induce C5b-9 release compared to the wild-type.

## Discussion

Complement activation is a hallmark of aHUS, C3 glomerulopathy and MPGN. Here we explored three patients with *CFB* variants. One of these variants, D371G, was shown to be a gain-of-function mutation, as indicated by enhanced binding to C3b, formation of the C3 convertase, increased hemolysis of rabbit erythrocytes and release of soluble C5b-9 from glomerular endothelial cells. Additionally, the D279G variant, also found in aHUS (13), was used a positive control and exhibited similar properties. Both *CFB* variants, D371G and D279G, could be effectively controlled by the FD small molecule-inhibitor danicopan (ACH-4471). The remaining two variants did not show gain-of-function but also did not perturb the inhibitory activity of danicopan. This suggests that FD inhibition should effectively inhibit complement activation in these patients.

*CFB* variants have been described in patients with aHUS (7–14, 31, 35, 36), in C3 glomerulopathy (15, 17) and in a few patients with immune-complex associated MPGN (16) but not all of them exhibit gain-of-function (12). Here we show that the mutant variant D371G, found in Patient 1, and reported previously (31), induces a clear-cut gain-of-function. These functional consequences can explain why the serum from Patient 1, without other complement mutations, induced C3 deposition on glomerular endothelial cells and hemolysis of sheep erythrocytes. Increased hemolysis in serum from this patient, treated with eculizumab at the time of sampling, is explained by the addition of rat serum, as a source of C5b-9, at which point the effect of eculizumab is eliminated by a washing step.

A novel *CFB* mutation E601K, in the serine protease domain of the protein, was found in Patient 2. This variant did not exhibit gain-of-function in the tests performed. Therefore, the increased complement deposition on endothelial cells cannot be explained by this genetic variant. The patient also had a mutation in the diacylglycerol kinase epsilon (DGKE) gene. DGKE mutations associated with aHUS do not directly cause complement activation and usually present during the first year of life (37), however this patient first presented with aHUS at mid-life. Thus, we assume that the DGKE variant was not associated with the patient’s disease.

The FB mutant variant I242L was detected in Patient 3, a child with what initially appeared to be immune complex-mediated MPGN. However, a second biopsy within 2 months showed more C3 deposition and suggested that the child might develop C3 glomerulopathy over time. This mutation was previously described in patients with aHUS and did not induce a clear gain-of-function (12). Serum from the patient induced C3 deposition on glomerular endothelial cells and enhanced hemolysis of sheep erythrocytes. The child also has previously reported genetic variants in *CFH*, V62I and N1050Y (38), suggesting that complement activation on cells may be a combined effect of *CFB* and *CFH* variants, although functional data regarding the *CFH* variant N1050Y are lacking.

One limitation of this study was that only three patients were investigated and only one of the three (Patient 1) was found to have a gain-of-function mutation in factor B (D371G). In Patients 2 and 3 we could not determine a link between the patients’ clinical disease and the factor B mutations. The absence of functional consequences of the two *CFB* mutant variants, E601K and I242L, is in apparent contradiction with the complement activation observed on endothelial cells, incubated with patient sera. Ex vivo complement activation on endothelial cells has been previously reported as positive in aHUS patients without identified genetic abnormalities (39). Moreover, it is positive in patients with sickle cell disease (40), preeclampsia and HELLP syndrome (41) and as shown herein, even in some apparently healthy controls. In sickle cell disease, the complement overactivation was mediated, at least in part, by heme (40, 42). Heme or other pro-inflammatory factors may be present in the patient sera, activating the endothelial cells, rendering them susceptible to complement activation. Furthermore, although serum from Patient 2 induced C3 deposition on endothelial cells the serum did not induce hemolysis (**Table 2**) which is in line with the presence of a pro-inflammatory factor inducing changes on the surface of endothelial cells which did not fully activate the terminal complement complex.

FD inhibition has been previously assessed in samples from patients with the complement-mediated diseases PNH and aHUS. Low concentrations of FD inhibitors were shown to reduce C3 fragment deposition on PNH erythrocytes as well as complement-mediated hemolysis (19, 20). Likewise, serum from aHUS patients induced complement-mediated cell death in *PIGA*-null PNH-like cells which was abrogated by the FD-inhibitor (20). The results of the current study focused on FB mutations utilizing both patient sera and recombinant mutants, showing that the FD inhibitor prevented FB cleavage to Bb, hemolysis and the formation of C5b-9 in the presence of gain-of-function mutations, thereby blocking excessive complement activation.

Danicopan was found to be effective in preventing complement-mediated hemolysis in a phase 2 trial in patients with PNH (43). A phase 3 trial is ongoing in which Danicopan is being investigated as add-on therapy to C5 inhibitor for patients with PNH with extravascular hemolysis (22). For patients with aHUS current consensus recommends treatment with intravenous eculizumab, or ravulizumab (5, 44) thereby blocking C5. FD inhibitors should also be evaluated in clinical trials either as add-on therapy for C3 glomerulopathy patients, or for aHUS patients in whom C5 inhibition is insufficient, or as an alternative therapy. FD inhibitors present certain advantages over eculizumab for the treatment of aHUS. In addition to the extremely high price of eculizumab (45) and the oral mode of administration of danicopan, FD blockade will selectively inhibit the alternative pathway and allow activity of the classic and lectin pathways. Patients treated with eculizumab are at risk of meningococcal infection due to blockade of C5b-9 mediated bacterial killing, a risk that is considerably less with FD inhibitors (46). However, C3 degradation fragments physiologically promote opsonization and phagocytosis (47) which are also important for defense against meningococcal infections. Inhibition of these proximal effects would not occur in the presence of eculizumab, while they would be impeded in the presence of an FD inhibitor. In line with this upstream inhibition, the spike protein of SARS-CoV-2 was shown to activate the alternative pathway of complement, and a small molecule FD inhibitor prevented the cellular deposition of C3 fragments and the generation of C5b-9 (48). Similarly, we could show that danicopan inhibited formation of the C3 convertase and FB cleavage as well as release of soluble C5b-9 from cells exposed to the gain-of-function *CFB* D371G mutation.

In summary, we describe at the molecular level, the response of FB mutations to FD inhibition and that FB mutations do not impact the effective response to FD inhibition. The data suggest that FD inhibition should be further studied in clinical trials as a possible treatment for complement-mediated kidney diseases aHUS, MPGN and C3 glomerulopathy.

## Data Availability Statement

The raw data supporting the conclusions of this article will be made available by the authors, without undue reservation, to any qualified researcher.

## Ethics Statement

The study of patients and healthy controls was performed with the approval of the Ethics Review Board at Lund University. The study was also approved by the National Bioethics Committee of Iceland and the Data Protection Officer at Oslo University Hospital, Oslo Norway. Informed written consent was obtained from the patients or the parents of Patient 3 and the healthy controls.

## Author Contributions

SA conceived and designed the analysis, analyzed the data; performed experiments and wrote the paper. A-CK performed experiments and wrote the paper. LR contributed conceptually, contributed plasmids and wrote the paper. AB contributed patient data and write-up. PK contributed patient data and write-up. RP contributed patient data, design and write-up. DK conceived and designed the analysis and wrote the paper. All authors contributed to the article and approved the submitted version.

## Funding

The Swedish Research Council (2017-01920), The Knut and Alice Wallenberg Foundation (Wallenberg Clinical Scholar 2015.0320), Skåne Centre of Excellence in Health, The IngaBritt and Arne Lundberg’s Research Foundation, Olle Engkvist Byggmästare Foundation (all to DK).

## Conflict of Interest

The authors declare that the research was conducted in the absence of any commercial or financial relationships that could be construed as a potential conflict of interest.
