# Supplementary figures and images for: Factor D Inhibition Blocks Complement Activation Induced by Mutant Factor B Associated With Atypical Hemolytic Uremic Syndrome and Membranoproliferative Glomerulonephritis
Source: Front Immunol. 2021 Jun 10;12:690821. doi: 10.3389/fimmu.2021.690821 (PMC8222914; doi:10.3389/fimmu.2021.690821)

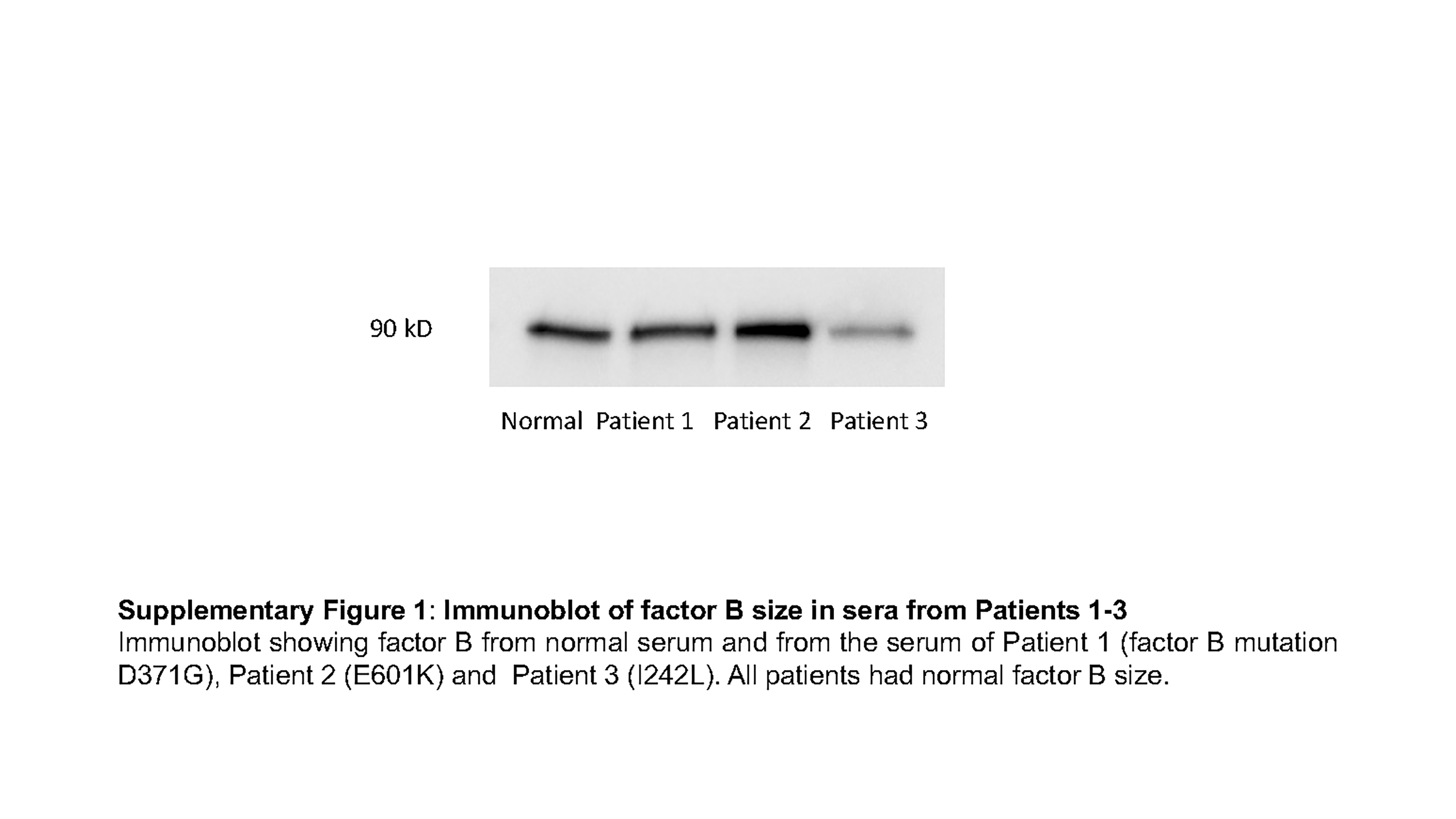

Supplement: Supplementary file 1 [file Image_1.tiff]
